# Shrinkage Characteristics of Geopolymer Concrete: A Comprehensive Review

**DOI:** 10.3390/ma18194528

**Published:** 2025-09-29

**Authors:** Rukayat Olayinka, Reza Jafari, Mathieu Fiset

**Affiliations:** Department of Applied Sciences, Université du Québec à Chicoutimi (UQAC), 555, Boulevard de l’Université, Saguenay, QC G7H 2B1, Canada; mathieu_fiset@uqac.ca

**Keywords:** geopolymer concrete, alkali-activated, shrinkage, pore structure, alkali concentration, curing condition, chemical additives, bio additives

## Abstract

Geopolymer concrete (GC) has become apparent as a promising and sustainable alternative to ordinary portland cement (OPC) concrete, presenting notable advantages in both environmental impact and mechanical performance. Despite these benefits, shrinkage remains a critical issue, influencing cracking susceptibility, long-term durability, and structural reliability. While previous investigations have focused on isolated parameters, such as activator concentration or curing techniques, this review provides a comprehensive analysis of the shrinkage behaviour of geopolymer concrete by exploring a broader range of influential factors. Key contributors—including precursor composition, alkali activator concentration, sodium silicate-to-sodium hydroxide ratio, liquid-to-solid ratio, pore structure, and curing conditions—are evaluated and mitigation strategies are discussed. Comparative evaluation of experimental studies reveals key patterns and mechanisms: heat curing around 60 °C consistently limits shrinkage, low-calcium binders outperform high-calcium systems, and chemical additives can reduce shrinkage by as much as 80%. The analysis also highlights emerging, bio-based additives that show promise for simultaneously controlling shrinkage and preserving mechanical performance. By integrating these diverse insights into a single framework, this paper provides a comprehensive reference for designing low-shrinkage GC mixtures.

## 1. Introduction

The global concern regarding greenhouse emissions and the drive for sustainability for the present and future generations has brought more attention to the construction industry, owing to its share of environmental pollution, accounting for 32% of global energy consumption and 34% of carbon emissions [1]. Despite sustainable practice awareness and implementations in recent years, activities of the construction industry have not been significantly affected when compared to the past decade reports of 31% and 39% of global energy consumption and carbon emissions, respectively [2]. Emissions are unlikely to decrease soon, as expanding infrastructure is necessary to accommodate the growing global population. Thus, investigating current construction materials and their sustainable alternatives is a key strategy to alleviate this issue.

**Figure 1:**
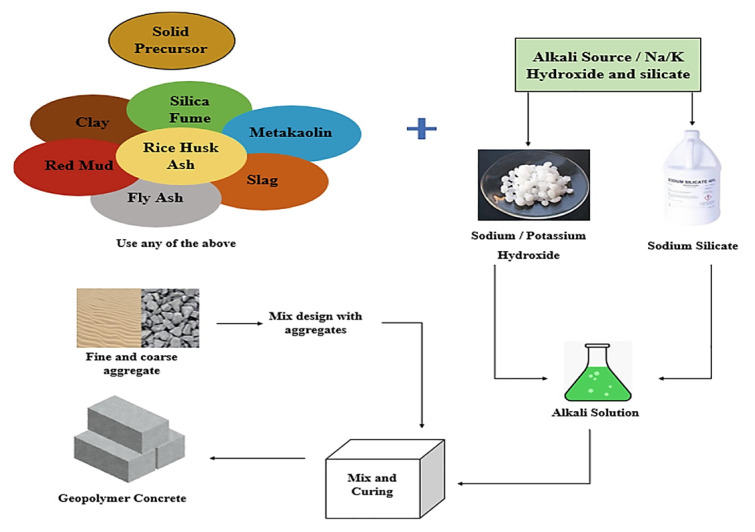
Process diagram for geopolymer concrete production [9].

Relative to concrete application in the construction industry, recent years have been followed by research focused on alternative materials for cement replacement in concrete due to its widespread use and the associated high environmental burden from its production (energy consumption and release of carbon dioxide), accounting for 5–8% of the total emissions [3,4]. Over the years, several waste and pozzolanic materials have been tested and found suitable to partially replace cement. However, the geopolymer concept has attracted wide international attention due to its potential as a full alternative to OPC concrete, offering enhanced engineering properties and environmental benefits [5,6,7]. Its manufacturing process is represented in Figure 1. Worthy of note is the recent publication of the first ASTM standard for geopolymer binders [8], a milestone that marks a key advancement in the field. By providing standardized testing, the reliability and regulatory recognition of geopolymer materials are enhanced, paving the way for broader industrial adoption and practical implementation.

Geopolymer concrete, one of the most advanced alternatives to OPC concrete, derives its superior properties from the type of binder material and activation process [10]. Geopolymer concrete was introduced in 1978 by Joseph Davidovits after experimenting on industrial byproducts, characterizing them as a class of mineral binders that resemble zeolites in their chemical makeup and amorphous microstructure [11,12]. It is mostly used in precast applications because it is relatively easy to handle delicate components (like high-alkali activating solutions) and because many contemporary geopolymer formulations demand a regulated high-temperature curing environment [12]. Various industrial by-products like fly ash, wood ash, slag, kaolin clay, fumed silica, and metakaolin can be used as base material for the geopolymer process [13,14], which makes it cost-effective and beneficial to the environment due to carbon footprint reduction [15]. Even though GC has demonstrated superior mechanical performance to OPC concrete [16,17,18], it may fall short in some other engineering aspects, like the shrinkage behaviour [19,20].

Shrinkage in concrete is a complex phenomenon that is affected by several variables, such as the mixture components, temperature and relative humidity of the surrounding air, and maturity of the concrete when exposed to the drying environment [21]. Shrinkage is a key durability concern in concrete technology, leading to cracking, loss of serviceability, and reduced structural performance. Both OPC and GC are susceptible to shrinkage; however, because of their different binder chemistries [22], the processes and degrees of shrinkage vary greatly. In OPC concrete, shrinkage primarily results from water evaporation, hydration reactions, and capillary tension in the pore structure [23,24]. However, GC shows a distinct shrinkage profile, which can be explained by a higher percentage of mesopores, which increases capillary stress during water loss, and a deficiency of calcium-based hydration products such as portlandite (Ca(OH)_2_), which can offer slight expansion and shrinkage compensation [20,25].

This paper provides an integrated understanding of shrinkage behaviour in geopolymer binders by considering a wide range of influencing factors, including precursor type and composition, alkali activator concentration, liquid-to-solid ratio, pore structure, and curing conditions. Unlike previous reviews that typically address one or two factors, this work synthesizes recent experimental findings to offer practical guidance for optimizing geopolymer formulations. Furthermore, it consolidates established mitigation practices and evaluates emerging low-shrinkage strategies, providing a technically grounded framework for the development of durable and sustainable geopolymer composites.

## 2. Geopolymer and Reaction Mechanism

### 2.1. Geopolymers

**Figure 2:**
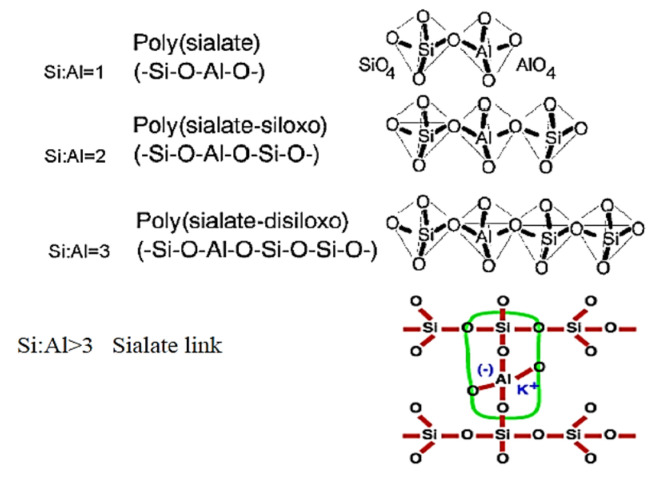
The polysialate formation of the geopolymerization process [28].

Geopolymers are inorganic, aluminosilicate-based materials that exhibit binding properties when activated by an alkali solution, forming a three-dimensional polymeric network through geopolymerization—a process in which silicon, aluminium, and oxygen atoms create a chain of SiO_4_ and AlO_4_ tetrahedra linked alternatively by shared oxygen atoms [26]. They form a network of polysialate with the general empirical formula:

(1) $$Mₙ\left[-{\left(SiO_{2}\right)}_{z}\;-\;AlO_{2}\right]ₙ\;\cdot \;wH_{2}O$$

where z is 1, 2, 3, or higher, *M* is a monovalent cation, such as potassium or sodium, *n* is the degree of polycondensation, and *w* is the number of available water molecules [27]. They are defined by three monomeric units (as shown in Figure 2) based on the Si/Al ratio, which highlights their durability, stability, and resistance to chemical attack in concrete [26].

Geopolymer application has gradually expanded into structural engineering due to promising results reported in numerous studies [10,28]. The geopolymerization process begins with the dissolution of aluminosilicate precursors (e.g., fly ash, metakaolin) in a highly alkaline solution, releasing silicate and aluminate species into the medium [29,30]. These species condense to form oligomers, which progressively polymerize into a three-dimensional aluminosilicate gel network [31]. The resulting gel solidifies through gelation, entrapping water and residual ions. Under certain curing conditions, this amorphous gel may partially reorganize into crystalline or semi-crystalline phases, potentially enhancing the mechanical and thermal performance of the material. These transformations are highly influenced by the physicochemical properties of the precursor materials, including their composition and reactivity [32].

### 2.2. Precursors

The raw materials needed for the geopolymerization process are usually rich in alumina and silica and can be found either naturally in clay, laterite, or similar materials or artificially in industrial waste products. The use of industrial by-products, such as fly ash, rice husk ash, and waste glass, not only enhances the sustainability of geopolymers compared to OPC but also leverages their high reactivity [10]. This reactivity is primarily due to the amorphous content of these materials, which allows aluminosilicate species to dissolve readily in alkaline solutions and participate effectively in the geopolymerization process. Materials with higher amorphous phases have been reported to produce durable geopolymer matrices with reduced shrinkage [33], making the selection of precursors with substantial amorphous content a critical factor in designing efficient geopolymer binders [34].

#### 2.2.1. Fly Ash

Fly ash (FA), also known as pulverized fuel ash, is a by-product of coal combustion and one of the most widely used precursor materials due to its high aluminosilicate content, availability, and cost-effectiveness, particularly in regions with extensive coal-fired power generation, such as China and India [35]. Its production process involves burning finely powdered coal in a boiler to create electricity. The ash is then captured in a power plant’s chimney using a particle control device (such as fabric filters or electrostatic precipitators). Mostly composed of glassy particles the size of silt and clay, FA has a consistency similar to talcum powder [36]. It should be noted that there are two classes of FA based on their chemical composition and source of coal combustion. Class F fly ash, which has a very low CaO percentage, is a common FA produced by burning bituminous coal. As new power sources, lignite and sub-bituminous coal are also utilized to generate class C fly ash, which has a high CaO concentration. In addition to its similarity to the composition of natural volcanic ash, class F fly ash offers the benefits of low cost, good spherical structure, richness in amorphous silicate, etc., and this makes it better for use as a geopolymer material [37].

#### 2.2.2. Ground Granulated Blast-Furnace Slag

This is a glassy granular substance that is created when molten blast-furnace slag is quickly cooled by submersion in water during the iron-making process in the steel industry. It is a non-metallic substance made up of silica, alumina, calcium oxide, and other bases that are molten alongside iron in the blast furnace [38]. Ground granulated blast furnace slag (GGBFS) is rich in both silicon oxide and calcium oxide, and with this composition, studies have shown that the strength achieved by GGBFS-based GC is similar to that of the OPC concrete [39].

#### 2.2.3. Metakaolin

Metakaolin (MK) is produced by heating kaolin, a natural clay mineral, to high temperatures—the calcination process—through the transformation of the crystalline kaolinite into an amorphous material [40]. In contrast to FA, silica fume, and slag, which are by-products of industrial processes, MK is one of the newly designed supplemental cementitious materials that is produced for a specific purpose under carefully regulated conditions [41]. However, the production of MK is relatively energy-intensive and costly, which, combined with its limited availability, raises concerns about its large-scale economic feasibility [42,43]. According to Sabir et al. [44], the calcining temperature of kaolin that produces the most active state of MK is usually between 600–800 °C. Above 850 °C, crystallization occurs and reactivity decreases. The dehydroxylation process of kaolin clay to form MK can be represented by Equation (2) [32]:

(2) $$Al_{2}Si_{2}O_{5}.{\left(OH\right)}_{4}\to Al_{2}O_{3}2SiO_{2}+2H_{2}O$$

#### 2.2.4. Bauxite Residue

Bauxite residue (BR), also known as red mud, is a type of hazardous solid waste by-product of the alumina manufacturing process with considerable alkalinity. It is characterized by its high-water content and intricate composition that varies with the bauxite composition, production process, dehydration, and storage time [45]. A study classified BR into three categories according to the alumina production process, as well as the quality of bauxite used: Sintered BR (SBR), Bayer BR (BBR), and Combined BR (CBR) [46]. SBR composition (including dicalcium silicate, calcium carbonate, and others) is similar to the basic ingredients used to fabricate OPC. Therefore, the most effective way to employ SBR is by incorporating it into the raw materials used to produce OPC, with utilization rates of roughly 95%, whereas BBR is the most popular refining technique [47]. It has high alumina and alkali content, making it inappropriate for direct incorporation into the OPC manufacturing process. However, studies have revealed that the Bayer method produces alumina with fewer impurities, superior product quality, a more efficient process, and lower production costs [46], with its resulting components rich in silica, alumina, and soda (alkali), demonstrating its advantageous potential for application in the manufacture of geopolymers [48].

#### 2.2.5. Alkali Activator

Alkali compounds (hydroxide and silicates) made of the first group metal elements in the periodic table, such as potassium (K) and sodium (Na), are the typical alkali activators that activate the geopolymer [49], for which sodium hydroxide (NaOH) and sodium silicate (Na_2_SiO_3_) are the most common due to their excellent rheology and inexpensive cost [50,51]. Alkali activators are highly caustic solutions and the fundamental component of geopolymers that trigger the pozzolanic reaction in aluminosilicate minerals. It is common knowledge that the alkali activation process is essential to polymerization kinetics, which involves turning solid aluminosilicate precursors into a hardened binder through an alkali reaction [52].

## 3. Shrinkage in Geopolymer Concrete

Like conventional concrete, shrinkage in geopolymer concrete is defined as the reduction in volume over time of the material at a constant temperature, and without any external loads [53]. This phenomenon is the main cause of cracking as the concrete hardens [54]. Geopolymer shrinkage occurs not only due to water loss over time from reaction (chemical consumption) and evaporation (free water in the pore network) but also because of the pore structure, which is influenced by key factors such as the alkali activator, water content, binder materials, and curing conditions [55,56,57]. Based on the mechanisms involved, the shrinkage in geopolymer concrete can be classified as plastic, autogenous, thermal, and drying shrinkage.

### 3.1. Plastic Shrinkage

Plastic shrinkage develops when wet concrete loses water through evaporation [58] or suction from an adjacent material, typically soil or underlying concrete. Specifically, it is the contraction of concrete while still in the semi-liquid form (plastic form) [54]. This could result in considerable cracking during setting and is typically affected by temperature, relative humidity, wind speed, binder content, and water-to-binder ratio. The use of ice or chilled water was suggested to reduce the initial concrete temperature, thereby reducing the water evaporation and plastic shrinkage [59].

### 3.2. Thermal Shrinkage

Thermal shrinkage is the volumetric contraction caused by temperature fluctuations, particularly following heat curing or during thermal exposure, as the material cools and stabilizes. Studies have reported that exposing hardened concrete to high temperatures (notably beyond 300 °C) causes changes in the geopolymer’s physicochemical properties [60,61], which can result in thermal shrinkage and macro-cracking. To manage mesoscale thermal deformations, it is important to retain the water content in the geopolymer mix [62]. A gradual cooling technique can also be employed after steam or heat curing to avoid thermal contraction. Furthermore, the type and composition of the alkali activator used play a crucial role in influencing thermal deformation. For example, the use of potassium as an alkali activator has been found to reduce thermal shrinkage more effectively than sodium [61].

### 3.3. Autogenous Shrinkage

Autogenous shrinkage is the self-desiccation of the geopolymer matrix, brought on by chemical reactions between the precursor materials and alkali activators, typically during setting and curing, which results in loss of capillary water, as well as an increase in capillary stress [63]. Despite the absence of external moisture loss, the concrete experiences substantial volume changes throughout the reaction processes. The autogenous shrinkage rate can, therefore, be minimized by reducing the reaction rate between the precursor and activator, which can be achieved by controlling the particle size of the precursor (coarser material with smaller surface area), lowering the temperature of the reaction (use of chilled water), using retarding agents to slow the process, etc. Control of pore size is also important, as smaller pores result in increased stresses and increased shrinkage [64].

### 3.4. Drying Shrinkage

Drying shrinkage is the volume reduction caused by the evaporation of internal pore water from the binder network during the drying process and often accounts for most of the total long-term shrinkage. When concrete deformation is restrained, drying shrinkage usually causes cracking. Even if it may not compromise structural integrity, this cracking generally leads to durability issues [65]. It is possible to decrease the drying shrinkage of concrete by using an additive that reduces the rate of shrinkage, which can also increase its resistance to cracking [59].

## 4. Factors Influencing Shrinkage in Geopolymer Concrete

### 4.1. Alkali Activator

Alkali activator has a direct influence on the geopolymerization process and the overall properties of the hardened geopolymer. Experimental results showed that geopolymer mortar specimens with a higher alkali content have reduced water absorption and apparent porosity compared to those with a lower quantity of alkali [66], all of which are factors that influence the shrinkage behaviour of the concrete. An increase in soluble silicate content in the alkali activator has been reported [67] to raise the Si/Al ratio in geopolymers, which helps reduce the volume of large pores and results in a more homogeneous gel. This reduction in large porosity minimizes the risk of micro-crack formation, enhances strength, and lowers permeability. Some researchers explained that the use of higher alkali, for example, using sodium-based solutions instead of potassium-based solutions, may help to reduce shrinkage [64]. This mechanism was attributed to the differences in the dissolution and reaction behaviour induced by the activator, which influences the pore size distribution and the kinetics of geopolymerization. Conversely, other researchers explained that a higher alkali solution concentration may lead to more rapid drying and shrinkage because it increases the degree of polymerization, resulting in a larger volume of gel [68]. The latter can be supported by a study [54] that reported that a decrease in alkali activator content decreases autogenous shrinkage. This implies that regulating the amount of alkali activators can enhance shrinkage performance by minimizing internal volume changes. Furthermore, a mixture of both alkali hydroxide and silicate is commonly used to improve concrete properties, balancing reactivity, strength, and workability while limiting shrinkage.

#### 4.1.1. Alkali Concentration

The dissolution rate of solid aluminosilicate rises with increasing alkali concentration, and the difference between the aluminium and silicon dissolution rates narrows [69]. Studies have reported that different pore-size distributions and deformation capacities are influenced by the alkali concentration and the activator modulus (defined as the molar ratio of SiO_2_ to Na_2_O in the activator solution), which leads to varying capillary pressures and drying shrinkage intensity [70]. In their study on the effect of alkali concentration [71], the researchers noted that an increase in sodium content in the activator from 4% to 8% caused drying shrinkage to be 3 to 3.6 times greater than that of OPC mortar for a constant activator modulus; and the drying shrinkage for sodium hydroxide and sodium carbonate-activated slag mortar increased with an increase in sodium concentration under the same conditions. Similarly, another investigation using a combined sodium-sulfate and sodium-silicate activator reported markedly higher autogenous shrinkage when the alkali concentration was increased to 2.75% with an activator modulus of 0.6, producing shrinkage values more than 50% greater than those at 1.75% with a modulus of 0.7 [72]. According to the authors, this behavior was attributed to increased refinement of the pore structure, which intensified capillary tension.

**Figure 3:**
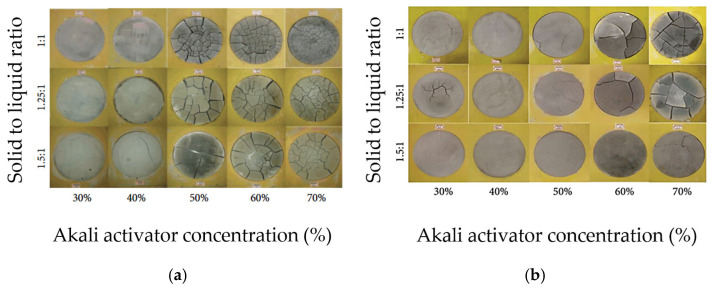
Top surface cracking caused by plastic shrinkage of GGBFS-based geopolymer paste according to different activator concentrations and solid to liquid ratio, for activator modulus (**a**) 1.5 and (**b**) 2.0 (from [68]).

**Figure 4:**
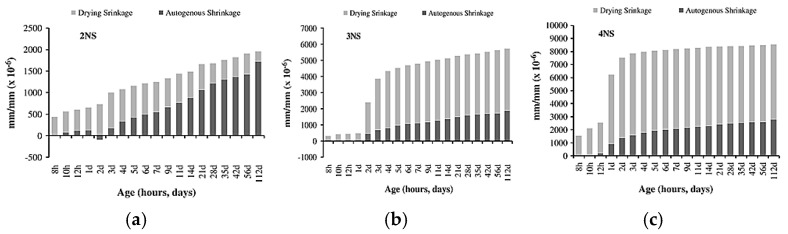
The autogenous and drying shrinkage results of GGBFS-based mortar with varying alkali concentrations of (**a**) 2NS (2.5% Na_2_O + 4.25% SiO_2_), (**b**) 3NS (3.5% Na_2_O + 5.95% SiO_2_), and (**c**) 4NS (4.5% Na_2_O + 7.65% SiO_2_) (from [73]).

Figure 3a,b show the top surface cracking of slag-based geopolymer pastes for different alkali concentrations of activator modulus 1.5 and 2.0, respectively. It can be observed that the number of cracks increases with increasing activator concentration. The authors explained that the dissolution and breakdown of aluminosilicates in slag are enhanced as the activator concentration increases, allowing alkali metal cations to strongly bind within the matrix. This reduces excess free ions and compacts the geopolymer microstructure. In addition, silicic acid, formed from sodium silicate hydrolysis, reacts with calcium hydroxide from slag hydration, producing small amounts of calcium silicate hydrate (C-S-H) gel. This disrupts the ionic balance, encouraging further calcium oxide dissolution from the slag, making the mix denser. While a higher activator concentration accelerates hydration and polymerization, an excessive alkali activator concentration can lead to excessive gel formation (greater than 50%), which significantly increases shrinkage and the likelihood of cracking. Melo Neto et al. [73] provided a similar explanation for their test results on slag-based alkali-activated mortar, demonstrated in Figure 4. The authors measured higher shrinkage values of the tested specimens when increasing the alkali (Na_2_O) concentration of the activator solution and explained that the higher amount of alkali activator promoted a faster hydration process, which resulted in the formation of more C-S-H gel. The increase in C-S-H gel volume densifies the pore structure, reducing the available space for water storage and movement. This makes the material more susceptible to internal tensile stresses during water loss, thereby contributing to higher shrinkage. Thus, a moderate alkali activator concentration of approximately 8–10 M is generally effective for managing shrinkage in geopolymers [74], although the optimal concentration is material-specific and should be confirmed by trial mixes.

#### 4.1.2. Liquid-to-Solid Ratio

**Figure 5:**
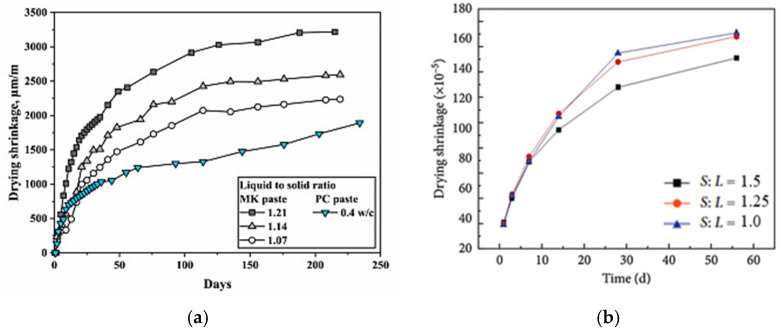
Effect of liquid-to-solid ratio on drying shrinkage of (**a**) MK-based geopolymer paste [76] and (**b**) GGBFS-based geopolymer paste (from [68]).

Several studies have investigated the effect of the liquid-to-solid ratio on geopolymer shrinkage. Results have shown that reducing the liquid-to-solid ratio can significantly reduce the geopolymer matrix shrinkage, similar to that observed for OPC concrete, where a lower water-to-binder ratio reduces shrinkage [75]. In an experiment to determine the effect of varying the liquid-to-solid ratio of an MK-based geopolymer paste, it was found that increasing the activator quantity (liquid) in the geopolymer mix increases the drying shrinkage of the paste. As shown in Figure 5a, the authors of this study stated that a decrease in the liquid-to-solid ratio from 1.21 to 1.07 could substantially minimize the drying shrinkage of the geopolymer by approximately 31%. The study by Xu et al. [68] supports this claim. The authors evaluated the effect of solid-to-liquid ratio on the shrinkage of GGBFS geopolymer paste using mixed sodium hydroxide and sodium silicate activator. They found that a decrease in the liquid-to-solid ratio is proportional to a decrease in the paste shrinkage. It was reported that the activator completely reacted with the slag due to the lower amount of liquid in the system, which enabled lower free water, and rate of water loss, resulting in less drying shrinkage (Figure 5b).

#### 4.1.3. Sodium Silicate to Sodium Hydroxide Ratio

**Figure 6:**
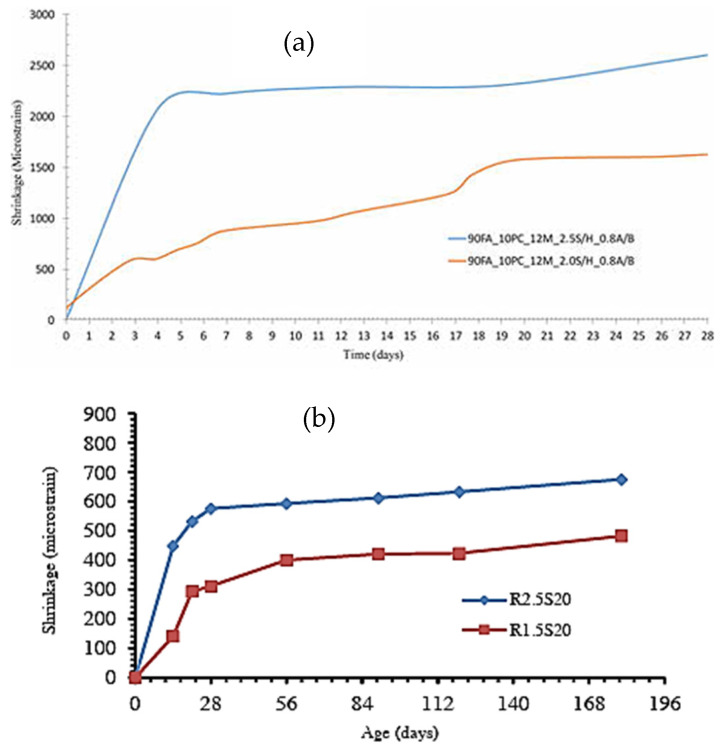
Shrinkage values of geopolymer mortars with varying sodium silicate to sodium hydroxide ratio (**a**) from [77], (**b**) from [51]. Specimens 90FA_10PC_12M_2.0S/H_0.8A/B and 90FA_10PC_12M_2.5S/H_0.8A/B in Figure 6a have a sodium silicate-to-sodium hydroxide ratio of 2.0 and 2.5, while specimens R1.5S20 and R2.5R20 in Figure 6b have a ratio of 1.5 and 2.5, respectively.

Different studies reported that an increase in the silica concentration of the alkali activator results in a notable increase in the shrinkage of the geopolymer composites [51,77]. The authors further stated that the higher self-desiccation of the geopolymer concrete may be connected to its refined pore structure, resulting from the high silica content associated with an increase in the activator modulus. Figure 6 shows the deformation caused by shrinkage of a fly ash-based geopolymer mortar (90% FA and 10% cement in Figure 6a and 80% FA and 20% GGBFS in Figure 6b). Results show that increasing the sodium silicate-to-sodium hydroxide ratio (SS/SH) leads to an increase in the shrinkage of the geopolymer mortar tested by these authors.

### 4.2. Binder Materials

Aside from water loss due to evaporation, the pore structure of geopolymers undergoes shrinkage as a result of several key factors, including the type of binder materials, alkali activators, water content, and curing conditions [55,78,79]. Among these, the binder’s initial characteristics, including molecular size, silica and alumina reactivity, and the presence of iron, calcium, and inert particles, play a crucial role [79]. It was reported that a reduction in the amount of geopolymer binder in a concrete volume can reduce its shrinkage [64]. The surface area and pore size distribution of the binder are also important factors influencing the extent of shrinkage. An experimental result [67] showed that substituting MK for FA in FA-based geopolymers can considerably increase the amount of water required to maintain the same workability, which is attributed to the high specific surface area of MK particles. Consequently, the high amount of water paves the way for high porosity and shrinkage, as previously discussed. Regarding the material composition, the calcium content in geopolymers can influence pore characteristics, particularly when high-calcium aluminosilicate precursors are used, as they promote the formation of C-A-S-H gel, which helps to fill the pores. But, in some cases, high calcium may cause self-desiccation (internal drying), increasing autogenous shrinkage and potential cracking [67]. Furthermore, C-A-S-H gels possess a comparatively low capacity to bind alkalis but a higher capacity to bind water than N-A-S-H gels [56,80]. As a result, mixtures containing larger amounts of C-A-S-H experience greater self-desiccation, since more water becomes chemically bound and less remains free, producing higher capillary tension in the matrix [81]. By contrast, Puertas et al. [82] found that binders rich in fly ash with less than 25% slag primarily form N-A-S-H gels, which hold less chemically bound water and consequently exhibit reduced tortuosity.

#### 4.2.1. Precursor Particle Size

**Figure 7:**
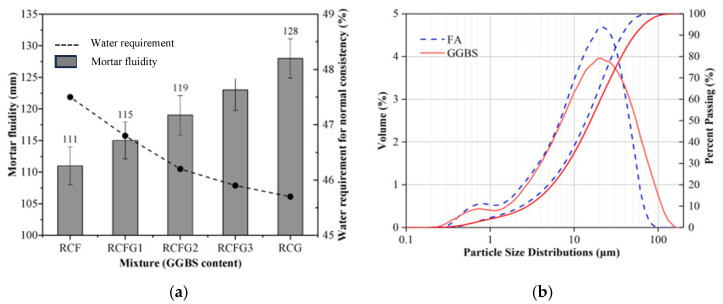
(**a**) Mortar fluidity and water requirement of geopolymer specimens for normal consistency, and (**b**) particle size distribution of FA and GGBFS (from [85]). RCF is the control mixture containing 100% fly ash (no GGBFS), and RCFG1, RCFG2, RCFG3, and RCG are the specimens prepared with the mixture containing 25%, 50%, 75%, and 100% replacement of fly ash with GGBFS, respectively.

Figure 7a presents the fluidity (evaluated by flow table test, see ASTM C 1437 [83]) and water demand for normal consistency (standardized flowability, see ASTM C 187 [84]) assessed for geopolymer binder specimens, all containing bauxite residue, but varying proportions of GGBFS and FA [85]. It can be seen in Figure 7a that the RCF (100% FA, 0% GGBFS) mixture achieved a flow value of 111 mm with a water requirement of 47.5%, whereas the RCG (0% FA, 100% GGBFS) achieved a flow of 128 mm with a water requirement of 45.7%. This indicates that increasing GGBFS content increases flowability and decreases water demand in the geopolymer mix. The authors explained that the decrease in water demand is due to the coarser nature of the GGBFS that was substituted for FA (see Figure 7b), which results in a less reactive surface that consumes less water. Some researchers also found in their investigation that a higher specific surface area of MK particles caused an increase in water demand when substituted with FA [67]. This increase in water demand can increase the amount of free water in the pores of the concrete structure, subsequently increasing shrinkage.

#### 4.2.2. Precursor Porosity

**Figure 8:**
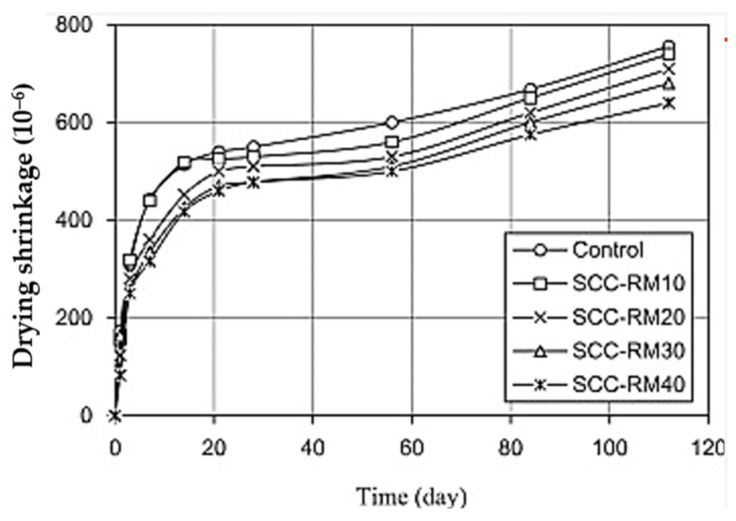
Drying shrinkage of self-compacting geopolymer concrete incorporating FA and BR. Specimens are labelled as SCC-RMXX, where XX indicates the percentage replacement of FA with BR (from [86]).

Liu & Poon [86] investigated the incorporation of BR to replace up to 40% FA in self-compacting geopolymer concrete (SCC) and found that BR considerably reduces the concrete mixture bleeding and segregation, thereby reducing its drying shrinkage. Figure 8 presents the drying shrinkage of the tested mixture with only FA (identified as Control) and the mixture with FA replaced by BR, identified as SCC-RMXX, where XX denotes the replacement percentage. As shown, the drying shrinkage of the SCC decreases with an increasing BR replacement percentage. According to the authors, this could be potentially explained by the internal curing capacity of BR. The porous nature of BR enables it to absorb a lot of free water while the concrete is still fresh. Then, as the concrete hardens, the water is gradually released from the BR to cure the concrete.

#### 4.2.3. Precursor Composition

**Figure 9:**
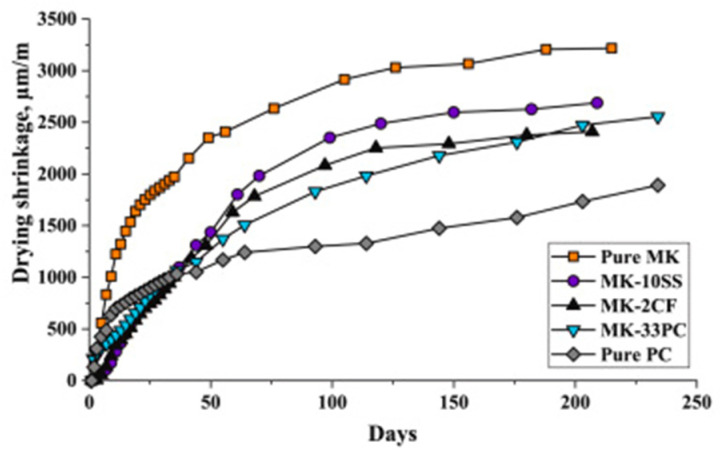
Drying shrinkage of various blends of geopolymer paste modified by GGBFS, carbon fibre and OPC, identified as SS, CF and PC, respectively (from [76]).

**Figure 10:**
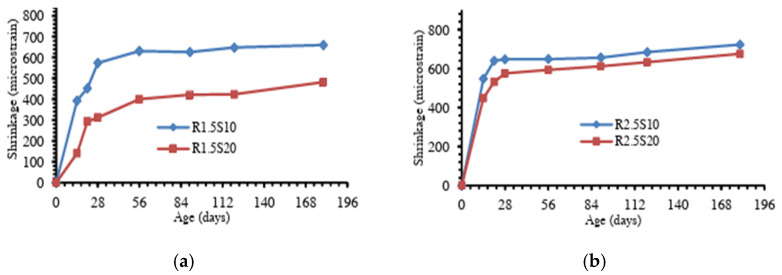
Drying shrinkage of FA-GGBFS blended geopolymer concrete with sodium silicate to sodium hydroxide ratios of (**a**) 1.5, and (**b**) 2.5 (from [51]).

Studies have shown that the chemical composition of the precursor influences the geopolymerization reaction kinetics, gel structure, and moisture retention of the geopolymer matrix [79,85]. In particular, the silica and calcium contents, along with the material surface area, are key factors, with their effects varying depending on the binder type. Although high calcium systems produce C-(A)-S-H gels that contribute to a denser matrix due to their relatively high Ca/Si ratios, the gels simultaneously refine the pore structure, generating higher internal stresses and capillary pressure, thus greater shrinkage [87,88]. In the case of low calcium binders, the high silica content promotes the dominant reaction to form fine N-A-S-H gels with low density [89], which affects the water movement within the matrix. In addition, a high surface area in precursor materials enhances reactivity, contributing to the formation of gels and influencing the shrinkage behaviour [67]. Blending different precursors can thus help optimize the binder composition and reduce concrete shrinkage. Figure 9 shows the results of different geopolymer pastes, blended with MK as the base material [76]. It was reported that after 200 days of curing, the equivalent drying shrinkages for geopolymer pastes modified by GGBFS, carbon fibre and OPC were approximately 16%, 25%, and 20% less than that of the non-modified paste. Calcium silicate hydrate (C-S-H) gel and ettringite were said to have been detected in the modified geopolymer products, which contributed to a more condensed matrix structure and reduced redundant water, thereby improving the paste shrinkage. Similarly, Figure 10 shows the result of a study on the drying shrinkage of GGBFS-blended FA-based GC [51]. Presented drying shrinkage tests were performed on geopolymer concrete at varying sodium silicate to sodium hydroxide ratios (identified as R1.5 and R2.5 for ratios of 1.5 and 2.5, respectively) up to 180 days, and in which FA was replaced with GGBFS at 10% and 20% composition (identified as S10 and S20, respectively). The results show that regardless of different sodium silicate-to-sodium hydroxide ratios, the shrinkage of both mixtures decreased by increasing the amount of GGBFS (or decreasing the amount of FA). However, the reduction in shrinkage is more pronounced in the lesser sodium silicate-to-sodium hydroxide ratio (mixtures R1.5).

### 4.3. Water-to-Binder Ratio

The higher the quantity of water in the concrete matrix, the higher the available pores. When these pores are interconnected, the permeability is typically high, leading to larger porosity and vice versa [55]. In OPC concrete, as well as geopolymer concrete, the water-to-binder ratio (w/b) is one of the key parameters influencing the matrix porosity and, thus, the concrete shrinkage. In GC, water facilitates the dissolution and transport of reactive species during geopolymerization and is partially consumed through physical entrapment within the gel network and evaporation during curing. When water content is insufficient relative to the binder, internal tensile stress can develop, increasing shrinkage and the risk of microcracking. On the other hand, a high w/b produces a matrix that is more porous, which encourages larger volume fluctuations during drying. This means that if the ratio is too high, it may also increase the drying shrinkage when extra moisture evaporates [90].

**Figure 11:**
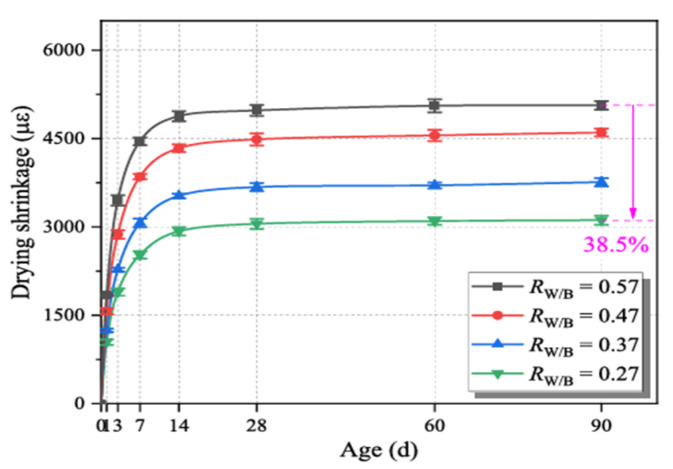
Drying shrinkage of various water-binder ratios (R_w/b_) from 0.27 to 0.57 of a blended geopolymer mortar made with FA, GGBFS, MK, and silica fume (from [91]).

Huang et al. [91] studied the effect of varying component ratios on the drying shrinkage of geopolymer mortar. The used precursor was a blend of FA, GGBFS, MK, and silica fume. Various w/b ratios were tested at 0.57, 0.47, 0.37, 0.27, using a mixture of sodium silicate and sodium hydroxide. The results presented in Figure 11 show that the shrinkage of the specimens decreased from 5063 με to 3113 με by reducing w/b from 0.57 to 0.27, which represents a shrinkage reduction of 38.5%. The authors proposed that water only served as a medium throughout the geopolymerization process, after which it moved from the sample’s interior to the surrounding environment due to the variation in relative humidity. According to the hypothesis of capillary water pressure (the movement of water within the spaces of a porous material due to the forces of adhesion, cohesion, and surface tension), the dissipation of water in capillary pores, particularly mesopores, induces considerable internal stress, which in turn leads to severe drying shrinkage when the water content is high.

### 4.4. Curing Conditions

The curing condition employed significantly influences shrinkage behaviour in GC because curing conditions affect moisture loss, reaction rate, and gel formation. High temperature curing (typically about 60 °C to 90 °C) tends to improve GC properties [74,92,93,94]. However, establishing standards for heat-curing systems is difficult, as it increases energy usage and thereby complicates practical implementation. The engineering application of FA-based geopolymers is still significantly hampered by curing temperatures; therefore, the curing technique employed depends on the priority property of the geopolymer application [95,96]. A study [97] reported that low-calcium FA geopolymers exhibit good performance under heat-curing conditions. However, fast polymerization induced by high curing temperatures might result in poor strength development and shrinkage cracks.

## 5. Mitigation Strategies for Shrinkage in Geopolymer Concrete

### 5.1. Chemical Additives

The addition of chemical additives (including expansive agents, shrinkage-reducing admixtures, superabsorbent polymers, and nanoparticles) has shown the most effective shrinkage-mitigating performance among various shrinkage-reducing strategies [53,98]. A compilation of various chemical additives and their respective roles in mitigating shrinkage in geopolymer composites is presented in Table 1.

**Figure 12:**
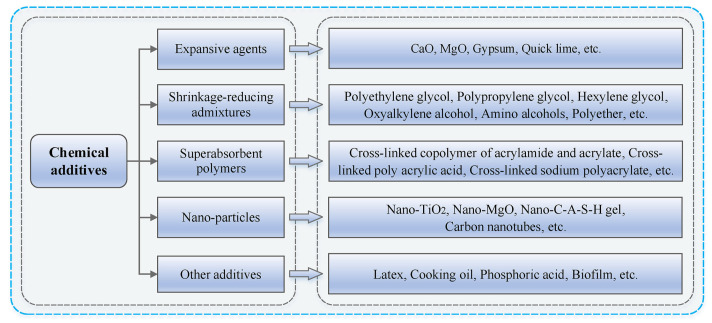
Classification of chemical additives used for geopolymer shrinkage mitigation [53].

Researchers explained that chemical additives can generally reduce shrinkage by lowering the surface tension and changing the pore structure of the geopolymer matrix [53], which in turn reduces the capillary tensile stress [54]. Numerous investigations have shown that increasing the amount of shrinkage-reducing admixture (SAP) in the material leads to a reduction in drying shrinkage [54,56]. Expansive agents have also been proven effective on geopolymer shrinkage reduction. A zinc-based expansive agent was highly effective at reducing shrinkage in geopolymers with a curing temperature of 80 °C and exposed to a confining pressure of 13.8 MPa [105]. Another study reported the effectiveness of nano-fibrillated cellulose with the combination of magnesium oxide expansive agent to mitigate the plastic and drying shrinkage of 3D printed GC [106]. Regarding super absorbent polymers, a study incorporated polyacrylamide, sodium polyacrylate, and sodium tetraborate, at 0%, 0.3%, 0.5%, 0.7%, and 0.9% to evaluate changes in the length and durability of GGBFS geopolymers and ascertain how the addition of polymer materials could reduce shrinkage [107]. According to the findings, 0.7% sodium tetraborate produced the minimal shrinkage value, recording 19% of the control value. The cracking pattern progression was recorded at 0 and 3 days of age using imaging technology, and results showed that polyacrylamide marginally reduced the crack propagation compared to the control group. The authors explained that these polymers can reduce shrinkage because they absorb and hold water, which they gradually release after the geopolymerization process. Figure 12 presents the different classifications of the chemical additives that can be used to mitigate shrinkage in geopolymer, as well as their prevalent examples.

#### 5.1.1. Expansive Agent

**Figure 13:**
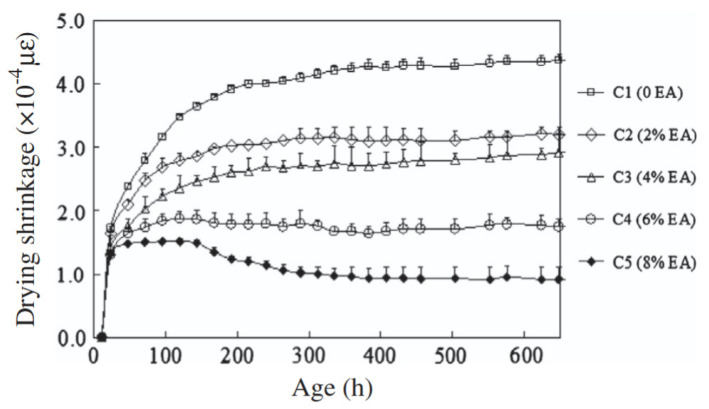
Effect of Expansive Agent (EA) on shrinkage of GGBFS-based geopolymer concrete. Specimens are labelled as CY, where Y represents the percentage of GGBFS in the binder composition, calculated as (100%–X), and X is the corresponding percentage of EA replacement (from [25]).

Figure 13 shows the 28-day results of the investigation of the effect of varying dosage of expansive agents (anhydrite and quicklime) on the shrinkage behaviour of GGBFS-based geopolymer concrete [25]. It can be deduced from the results that expansive agents can significantly reduce shrinkage in geopolymer concrete. The authors stated that the compensating mechanism for the shrinkage arose from the formation of portlandite Ca(OH)_2_, in the matrix. Thus, the higher the dosage of the expansive agent, the lower the drying shrinkage of the concrete. However, they noted that an increase in the dosage of the expansive agent above 8% may lead to concrete with expansive behaviour, rather than compensating for shrinkage.

#### 5.1.2. Shrinkage-Reducing Admixture

**Figure 14:**
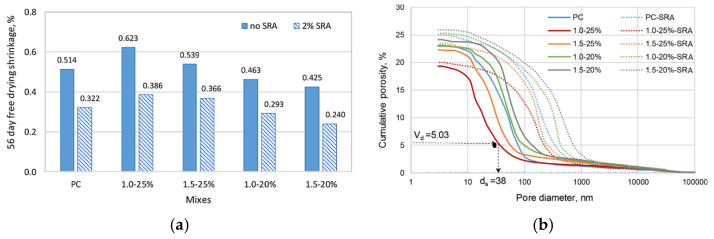
Effect of SRA on (**a**) shrinkage and (**b**) porosity of FA-based geopolymer pastes with activator moEffect of SRA on (**a**) shrinkage and (**b**) porosity of FA-based geopolymer pastes with activator modulus of 1.0 and 1.5, and activator concentrations of 20% and 25%. The OPC reference paste is labelled as PC, while the FA-based geopolymer pastes are labeled as XX–YY%, where XX denotes the activator modulus and YY the activator concentration (from [55]).

Various types of shrinkage-reducing admixtures (SRA) have been tested on geopolymer mixtures by authors in the literature [98,100]. Hexylene glycol-based SRA was used to investigate the shrinkage behaviour of FA-based geopolymer paste, and results were compared to OPC paste [55]. Figure 14 illustrates the 56-day shrinkage behaviour of both OPC and geopolymer pastes, including mixtures with 2% SRA. As seen in Figure 14a, despite variations in mixture composition, the addition of SRA reduced the 56-day shrinkage of all geopolymer pastes. The authors attributed the reduction in shrinkage not only to the lowered surface tension of water within the pore network but also to alterations in the pore structure induced by the SRA, as presented in Figure 14b, which shows a shift of the cumulative porosity curve from left (smaller pores) to right (larger pores), indicating an increase in the coarseness of the pore after the addition of SRA.

**Figure 15:**
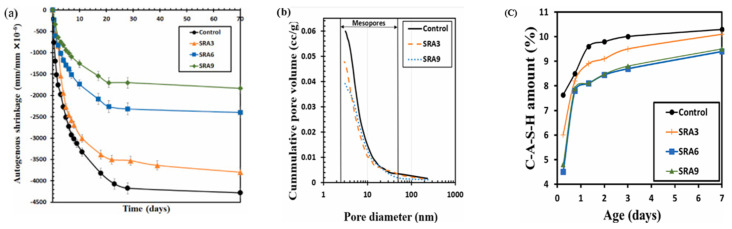
Shrinkage-reducing admixture (SRA) effect on (**a**) shrinkage, (**b**) pore volume, and (**c**) gel formation of a GGBFS-based geopolymer paste and comparison to a control paste without SRA (from [98]).

A recent research on the same subject explained that the refinement of the pore structure is part of the driving mechanism of SRA in reducing autogenous shrinkage in a GGBFS-based geopolymer matrix [98]. The researchers studied the shrinkage, porosity, and amount of C-A-S-H gel, and their results presented in Figure 15 also showed that the addition of 3%, 6%, and 9% glycol ether-based SRA (mixtures SRA3, SRA6, and SRA9, respectively) reduce shrinkage compared to a reference mixture without SRA. However, the cumulative pore structure presented in Figure 15c slightly shifted from right to left, indicating an increase in the pore fineness property rather than coarsening, as observed by [55] for FA-based geopolymer (Figure 14b). Furthermore, the reduction in the pore structure of the concrete was directly related to a reduction in the formation of the C-A-S-H gel (Figure 15b) and, by extension, a decrease in the gel porosity.

#### 5.1.3. Superabsorbent Polymers

**Figure 16:**
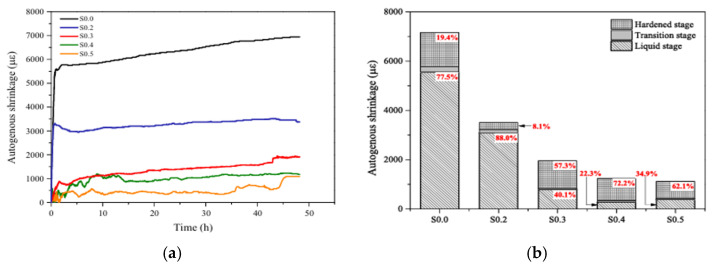
Effect of superabsorbent polymers dosage (0% to 0.5%) on autogenous shrinkage of fly ash-GGBFS-based geopolymer paste: (**a**) progression from 0 to 48 h, and (**b**) its final value (48 h) according to the geopolymerization stage, (from [101]).

Superabsorbent polymers (SAP) derive their ability to reduce shrinkage in geopolymer materials through the mechanism of internal curing [102,108]. They are reported to absorb excess of free water from the mixture and then release it into the composites during the hydration phase, which maintains a high level of internal relative humidity of the matrix [109,110]. Figure 16 presents the effect of the SAP dosage (identified as SXX, where XX referred to the dosage) on the autogenous shrinkage of fly ash-GGBFS-based geopolymer paste. A cross-linked copolymer of acrylamide and potassium acrylate SAP was incorporated in the geopolymer paste in dosages ranging from 0 to 0.5%, and the autogenous shrinkage was measured during the first 48 h. The results indicate that the tested amount of SAP is effective in reducing shrinkage. Dosages up to 0.3% of SAP (specimen S0.2 and S0.3) produce the most significant reduction in shrinkage compared to a reference mixture without SAP (specimen S0.0). However, increasing the SAP dosage above 0.3% (specimens S0.4 and S0.5) has a less significant effect on the shrinkage [101]. The authors explained that the shrinkage reduction can be tied to the reduction of capillary pressure resulting from the internal curing (supplementing moisture loss) effect of the SAP, which subsequently reduced the self-desiccation of the paste.

### 5.2. Material Optimization

Optimization of the raw materials can effectively reduce shrinkage in GC by improving the reaction process and pore structure. A study on FA geopolymer mortar shrinkage [95] revealed that the drying shrinkage decreases significantly as the OPC and silica fume (SF) content added to the binder increases compared to a 100% FA reference mixture. The samples with 30% replacement (20% OPC + 10% SF) exhibited 60.89% reduction in porosity. According to the authors, the reduction is attributed to the incorporation of OPC and SF, which enhances polymerization, accelerates the reaction process, and facilitates the formation of both C-A-S-H and N-A-S-H gels. These gels contribute to pore filling, minimize volumetric changes, and reduce shrinkage in FA-based geopolymer mortar. This finding contrasts with the earlier report on GGBFS-based geopolymer paste (Figure 15), where shrinkage reduction was associated with a decrease in C-A-S-H gel formation, highlighting that different mechanisms occur depending on the material type. The authors further stated that incorporating OPC and SF effectively controls drying shrinkage, resulting in improved interfacial bond strength and reduced porosity and permeability in geopolymers. However, excessive incorporation of these supplementary materials can create mix-design incompatibility and compromise other performance properties. For example, replacing silica fume at levels up to 10% of the binder has been reported to lower workability [111] and, in some cases, reduce strength [112]. Likewise, adding OPC can accelerate setting and alter workability [113]; and its calcium-rich phases make the matrix more susceptible to sulfuric-acid attack, where gypsum formation promotes cracking and durability loss [4,114].

While some studies report that partially blending calcium-rich precursors with low calcium binders reduces shrinkage [33,51,115], others have shown that incorporating fly ash into GGBFS-based geopolymers also improves shrinkage performance [116,117]. The literature established that shrinkage is generally greater in high-calcium geopolymer systems than in low-calcium ones. Drying shrinkage has been reported to diminish, up to 20–25% replacement of low calcium binders with GGBFS [51,82]. When this replacement exceeds 30% by weight, the mixtures tend to show higher alkali efflorescence and greater shrinkage [118,119,120]. Similarly, optimizing the alkali activator, considering the factors discussed earlier [68,76,77], can help mitigate shrinkage. For instance, the activator modulus plays a key role in balancing strength and shrinkage. Adequate silicate content is essential for forming a strong aluminosilicate network and promoting gel formation; however, excessive gel can restrict water mobility and accelerate capillary pressure development [121]. Most studies, therefore, maintain a modulus within the range of 1–1.5 to achieve a balance between strength and shrinkage. Table 2 provides a summary of various blended precursor materials used in geopolymer systems, highlighting their influence on shrinkage reduction. Likewise, Table 3 presents an overview of different alkali activators employed in geopolymer studies, emphasizing the effectiveness of raw material optimization and balance in shrinkage mitigation.

### 5.3. Curing Conditions

The curing conditions, duration, and technique play a major role in shrinkage. Compared to heat-cured specimens (temperature of 60 to 90 °C), ambient-cured specimens (temperature of 20 to 25 °C) exhibit higher drying shrinkage [126,127]. Experimental results from a study indicated that steam-cured GC experiences enhanced condensation reactions, leading to faster capillary moisture loss and a more complete reaction process, as evidenced by the rapid early strength development observed [65]. As reported by the authors, very low shrinkage of less than 400 με after 1 year (below the Australian limit of 700 με [128]) was measured for the steam-cured GC specimen compared to the corresponding control specimen of 500 με.

This can be supported by another study that investigated the effect of three curing methods on drying shrinkage of fly ash-based GC [129]: curing for 3 days at ambient temperature, dry heat curing for 24 h at 60 °C, and steam curing for 24 h at 60 °C. After 3 months, the drying shrinkage of the ambient-cured specimens was around 1300 με, which, according to the researchers, is roughly two-to-three times greater than what would be expected for equivalent OPC concrete. On the contrary, heat and steam-cured GC specimens of variable mixtures performed exceptionally well, exhibiting shrinkage of approximately 100 με or less after 1 year. The authors explained that most of the water released after the initial consumption by the chemical reaction may evaporate due to the heat. Because little water is left in the hardened concrete’s micropores, there is also relatively little induced drying shrinkage as opposed to the ambient curing condition.

Few studies state that heat curing reduces the shrinkage of GC concrete. A study investigated the impact of curing temperature and duration on shrinkage control [94]. It was reported that an FA-based GC required a minimum of 3 days when cured at 40 °C or 1 day at 80 °C to achieve drying shrinkage values comparable to or below maximum values specified for structural concrete in Eurocode 2 (EN 1992-1-1) [130], while extending 80 °C cure to 7 days has little effect on the drying shrinkage, although it had a more significant impact on the GC compressive strength. Similarly, another study showed that GC heat-cured at 90 °C for 24 h showed lower early-age shrinkage than OPC concrete (about 66% of OPC value at 21 days), emphasizing the effect of heat curing on FA-based geopolymer composites [131]. Results from other researchers also confirmed a decrease in the autogenous shrinkage of an FA-based geopolymer slurry from 2.63% to 2.21% when the temperature increased from 40 °C to 80 °C, following a 120-h reaction [132].

On the contrary, some researchers reported that shrinkage in GGBFS-based geopolymer composites increased with heat curing. A study noted that the autogenous shrinkage of a GGBFS-based geopolymer mortar cured at 60 °C increased up to 3430 με in 14 days, which is about 18% above the same specimen and cured at 20 °C, with a shrinkage value of 2890 με [93]. This is also supported by another study that investigated ambient and oven-dried samples of FA-GGBFS blended geopolymer concrete [124]. Results showed that oven-cured specimens at 60 °C exhibited higher drying shrinkage compared to the ambient-cured specimens. The authors further investigated the effect of increased curing temperature and found that the shrinkage in GC increased with increasing curing temperature, from 60–120 °C. Table 4 presents various curing systems investigated over time and their respective roles in reducing shrinkage in geopolymer composites.

Carbonation curing is another technique explored for geopolymer composites, although not commonly adopted compared to conventional thermal or ambient curing methods. Studies have shown that carbonation curing decreases GC porosity while simultaneously increasing strength [95]. This decrease in porosity might be the result of CO_2_ seeping into the mortar matrix during the carbonation curing process, where it takes part in the hydration reaction to generate CaCO_3_ and Na_2_CO_3_, which fill the specimens’ surface pores. By preventing the loss of internal moisture, these plugged pores guarantee that there is enough water for hydration and, consequently, less shrinkage. However, prolonged carbonation curing might lead to matrix deterioration and increased drying shrinkage.

Overall, heat curing has been reported to be more effective in most studies, with about 30–60% shrinkage reduction [134,135]. A curing temperature of 60 °C is recommended, as it provides a balance between strength development and shrinkage; higher curing temperatures may increase shrinkage rather than mitigate it. In addition, the recommendation aligns with the standardized temperature for elevated curing in alkali-activated cementitious materials [8].

## 6. Emerging Strategies

Research on GC shrinkage has traditionally emphasized the roles of precursor chemistry, alkali activators, and curing conditions as discussed above. While shrinkage reduction is desirable, varying these factors often interacts with other key properties, particularly mechanical performance [136], such that in applications demanding high strength, shrinkage resistance may be compromised. Chemical additives, on the other hand, have been proven to reduce shrinkage up to 80% [53,137], with effective ranges typically between 0.3–0.5% of binder weight. However, it has also been reported to have some negative impacts on other properties of GC. For instance, a concrete sample treated with two different SAPs saw an approximate 10% reduction in compressive strength in both additives, albeit with improved shrinkage [137]. Similarly, the use of SRAs has been reported to compromise mechanical performance in certain shrinkage reduction studies [138,139,140], with comparable trends also observed for expansive agents [141]. Therefore, there is a growing need to shift attention beyond the constituting materials of the geopolymer and chemical additives, towards additives (e.g., bio-additives) that can mitigate shrinkage without undermining durability. This perspective introduces the requirement that not only must shrinkage be controlled, but the additive must also maintain the strength, durability, and sustainability advantages that underpin the motivation for adopting geopolymer systems in the first place.

For example, some researchers found that the incorporation of vegetable oil into an MK-GGBFS geopolymer significantly reduced drying shrinkage. Under stationary conditions at 65% RH, the oil-modified system exhibited approximately 50% lower drying shrinkage compared to the control. The authors attributed the improvement to the formation of soap phases that filled gel and fine capillary pores (<20 nm), leading to a substantial decrease (64%) in open porosity. The addition of oil resulted in mixed mechanical performance, showing both reductions and improvements depending on the relative humidity conditions [142]. The influence of starch-derived bio-based plasticizers on the shrinkage and mechanical behaviour of MK-based geopolymer mortars was also investigated by some other researchers. They found that at a maximum dosage of 1%, the additive resulted in an approximate 47% reduction in shrinkage after 56 days, a 40% increase in slump, an approximately 64% increase in 28-day flexural strength, and about a 2% increase in compressive strength [143]. Some other bio-additives (natural sugars and terminalia chebula) have also been reported to improve the physico-chemical properties of FA-GGBFS-based self-cured geopolymer mortars [144]. All of these point to the feasibility that bio-additives can be a better and sustainable solution to shrinkage in GC.

## 7. Conclusions

Although shrinkage in geopolymers is well understood, it remains a significant limitation for practical applications due to its multifactorial phenomenon, influenced by several factors, including precursor composition, activator chemistry, water content, curing conditions, and chemical additives. This paper highlights mitigation strategies to reduce shrinkage, with the most effective approach depending on the specific geopolymer composition and intended application. Based on the reviewed studies, it can be concluded that:

(1) The activator concentration and the liquid-to-solid ratio are dominant factors, with greater values leading to increased shrinkage due to higher gel volume, denser capillary networks, and intensified drying stresses.
(2) Reducing the water-to-binder ratio generally reduces drying shrinkage by minimizing pore formation and limiting water evaporation. A lower w/b ratio results in a denser matrix with reduced capillary stresses, while higher ratios increase porosity and moisture loss, leading to greater drying shrinkage.
(3) Precursor composition, particularly calcium content, plays a crucial role; calcium-rich systems can form C-A-S-H phases that may partially mitigate shrinkage in low calcium binders.
(4) High-temperature curing (oven and steam, at approximately 60 to 80 °C) typically reduces shrinkage due to accelerated reaction kinetics and early matrix stiffening, while ambient curing prolongs the shrinkage-active period due to slower polymerization and, thus, greater exposure to drying and shrinkage. Nonetheless, some studies reported that excessive thermal curing may increase shrinkage in GGBFS-based systems, underscoring the need for optimized curing protocols.
(5) Chemical additives show great potential for mitigating shrinkage when used in appropriate dosages. These additives reduce the internal surface tensile stress and refine the pore structure of the geopolymer matrix.
(6) Bio additives show promising mitigation potential in balancing shrinkage behaviour and the mechanical performance of geopolymer composites.
(7) Most studies found that geopolymer composites generally shrink more than OPC composites. However, the mitigation strategies presented can reduce shrinkage in geopolymer composites below the values typically observed for OPC.

## 8. Future Directions

Shrinkage investigation in geopolymer composites needs to move beyond ambient drying conditions to account for realistic environmental exposures. In particular, shrinkage under freeze–thaw cycles in cold regions and early-age behaviour in emerging applications such as 3D printing remain relatively under-explored. Equally important is the further investigation of the effects of bio-based additives on shrinkage and mechanical properties. Future research should also examine the fire and high-temperature performance of these materials [145], to better understand their thermal stability and structural integrity under extreme conditions. Addressing these directions not only fills critical performance gaps but also aligns with the broader goal of developing sustainable and resilient geopolymer concretes.

## Figures and Tables

**Table 1:** Summary of the influence of chemical additives on geopolymer shrinkage.

Matrix Type	Additive Type	Dosage by Mass of Binder	Precursor 1. FA 2. GGBFS 3. OPC	Activator 1. NaOH 2. Na_2_SiO_3_	Modulus	Alkali Concentration	Activator/ Binder Ratio	Curing Condition	Age	Type	Shrinkage Value (με)	Ref.
Concrete	EA (Anhydrite and quick lime)	0% 2% 4% 6% 8%	2	1 + 2	1.5	10%	0.58	20 ± 3 °C, 60 ± 5% RH	28 d	Drying	430 320 290 170 90	[25]
Mortar	EA (MgO powder)	0% 3%	2	1 + 2	0 0.5 1.0 1.5	4%	-	Cured at: 20 ± 1 °C 95 ± 3% RH Dried at: 20 ± 1 °C 50 ± 3% RH	56 d	Drying	Dosages 0% 3% 1700 2100 2500 2300 4100 3150 5200 3300	[99]
Mortar	SRA (Tetraguard AS21, BASF)	0% 3%	2	1 + 2	0 0.5 1.0 1.5	4%	-	Cured at: 20 ± 1 °C 95 ± 3% RH Dried at: 20 ± 1 °C 50 ± 3% RH	56 d	Drying	Dosages 0% 3% 1700 900 2500 1300 4100 1500 5200 3400	[99]
Paste	SRA (Hexylene glycol)	0% 2%	1-class C	1 + 2	1 1.5	20% 25% 20% 25%	0.33	Sealed (7 days) at: 50 ± 2 °C 55% RH Dried at: 23 ± 2 °C 50 ± 4% RH	56 d	Drying	Dosages 0% 3% 0.46% 0.29% 0.62% 0.39% 0.43% 0.24% 0.54% 0.37%	[55]
Mortar	SRA (Ether glycol)	0% 3% 6% 9%	2	1 + 2	1	8 M	0.4	Sealed with plastic foil	70 d	Auto	4200 3700 2400 1800	[98]
Mortar	SRA (Polypropylene glycol)	0% 1% 2%	3 2 2 2	1 + 2	1–1.2	4% Na_2_O	0.42 0.58 0.55 0.5	20 ± 2 °C, 99% and 50% RH	180 d	Auto and Drying	RH 99% 50% 0.02% 0.12% 0.11% 0.44% 0.06% 0.40% 0.03% 0.28%	[100]
Paste	SAP (copolymer of acrylamide and potassium acrylate)	0% 0.2% 0.3% 0.4% 0.5%	1-class F (75%) + 2 (25%)	1 + 2	2.58	10 M	0.4	Sealed Dried at: 20 °C, 40% RH	48 h	Auto	7200 3600 2000 1300 1200	[101]
Paste	SAP (sodium polyacrylate)	0%, 0.3%	2	1 + 2	0.8 1 1.4	4% Na_2_O	-	-	168 h	Auto	Dosages 0% 0.3% 4400 750 5000 1300 4700 1200	[102]
Mortar	NP (nano-C-A-S-H gel)	0% 1% 3% 5%	2	2	1.8	-	-	Cured at: 20 ± 1 °C 90% RH Dried at: 20 ± 1 °C 50 ± 3% RH	60 d	Auto and Drying	Auto. Drying 2350 2900 1700 2000 1600 1900 1800 2500	[103]
Paste	NP (nano-TiO_2_)	0% 1% 3% 5%	1	1 + 2	1.5	10 M	0.5	Dried at: 20 ± 3 °C 90 ± 5% RH	28 d	Drying	1050 950 670 550	[104]
EA—Expansive agent, SRA—Shrinkage reducing admixture, SAP—Super absorbent polymer, NP—Nano particle												

**Table 2:** Summary of the influence of raw materials on geopolymer shrinkage.

Matrix type	Main Precursor 1. FA 2. GGBFS 3. MK 4. Rice husk ash 5. OPC	Additional Precursor 1. FA 2. GGBFS 3. BR 4. OPC 5. Silica fume	Activator 1. NaOH 2. Na_2_SiO_3_	Alkali Concentration/Molarity	Activator/ Binder ratio	SS/SH ratio	Modulus	Age	Type	Remark	Ref.
Concrete	4	2 (ultra fine)	1 + 2	8 M	0.35	2.5	1.48	90 d	Drying	Negative Increased shrinkage, although with an insignificant value	[115]
Concrete	1 + 5	3	-	-	0.5	-	-	110 d	Drying	Positive Reduced shrinkage, possibly due to the internal curing effect of BR	[86]
Concrete	1	2 (20%, 25%)	1 + 2	14 M	0.4	1.5 2.5	2.61	180 d	Drying	Positive Shrinkage reduced with increasing slag content, irrespective of SS/SH ratio	[51]
Paste	3	2 and 4	1 + 2	-	1.2	-	1–2.5	200 d	Drying	Positive Shrinkage improved with additional precursor material blend, with a 16% and 20% shrinkage reduction for 10% slag and 33% OPC, respectively	[76]
Mortar	2	1 and 5	1 + 2	5% Na_2_O	0.35–0.425	-	1.2	120 d	Drying	Positive Shrinkage improved with each additional precursor. Although the mix blend with silica fume has a more pronounced shrinkage reduction compared to that of fly ash.	[122]

**Table 3:** Summary of the influence of alkali activators on geopolymer shrinkage.

Matrix Type	Precursor 1. FA 2. GGBFS 3. OPC	Activator 1. NaOH 2. Na_2_SiO_3_ 3. Na_2_CO_3_ 4. Na_2_SO_4_	Influencing Factor 1. Liquid to Binder Ratio 2. Activator Concentration 3. SS/SH Ratio 4. Activator Type	Concentration	Activator/ Binder Ratio	SS/SH Ratio	Mod-Ulus	Curing Condition	Age	Shrink-Age Type	Shrinkage Value (με)	Ref.
Mortar	3 2	- 1 2 3	4	8% Na_2_O	0.5	-	0.75	20 ± 2 °C, 65 ± 5% RH	6 M	Drying	0.1004% 0.2968% 0.3642% 0.1053%	[71]
Paste	2	1 + 2	1	40%	1 0.8 0.67	-	1.5	Cured: 20 °C, 95% RH Dried at ambient conditions	56 d	Drying	1510 1600 1710	[68]
Paste	2	1 2 4 1 + 2 + 4 1 + 2 1 + 4 2 + 4	4	6% Na_2_O	0.4	-	1.5	20 ± 2 °C, 95 ± 5% RH	14 d	Auto	2300 6500 1500 2000 5200 1750 2800	[123]
Mortar	1	1 + 2	2	6 M 8 M 10 M	0.5	2.5	-	Sealed, oven cured (24 h at 60 °C) Dried at: 25 °C, 50% RH	90 d	Drying	1000 850 800	[74]
Concrete	1 + 2	1 + 2	1	10% Na_2_O	0.4 0.45 0.5 0.55 0.6 0.65 0.7	-	-	Ambient and oven-cured for 24 h at 60 °C	28 d	Drying	Ambient Oven 354 415 365 430 380 465 392 485 405 495 420 523 435 533	[124]
Paste	1 + 2	1 + 2	2	Na_2_O 4% 5% 6% 7% 8%	0.38	-	1.2	Cured (3 d) at: 20 °C, 95% RH Dried at: 20 ± 2 °C, 55 ± 5% RH	90 d	Drying	15,000 12,400 12,000 9300 9500	[70]
Mortar	1	1 + 2	3	10 M	0.5	0.5 1.5 2.5	-	Sealed, oven cured at 24 h-60 °C Dried at: 25 °C, 50% RH	90 d	Drying	800 950 990	[74]
Paste	2	1 + 2	2	NaOH 30% 40% 50% 60% 70%	1.5	-	1.5	Cured at: 20 °C, 95% RH Dried at ambient conditions	28 d	Plastic	Block per piece 7 5 22 50 85	[68]
Concrete	1 + 2 (10%) 1 + 2 (20%)	1 + 2	3	14 M	0.4	1.5 2.5	2.61	Sealed: 7 days Dried at: 20 ± 2 °C, 70 ± 10% RH	180 d	Drying	10% 20% 680 510 720 700	[51]
Concrete	4	1 + 2	2	8 M 14 M	-	-	-	Ambient	28 d	Drying	8 M 14 M 42 41	[125]

**Table 4:** Summary of the influence of curing techniques on geopolymer shrinkage.

Matrix Type	Curing Type	Precursor 1. FA 2. GGBFS 3. OPC 4. Kaolinite Ash 5. Glass Powder 6. Silica Fume	Activator 1. NaOH 2. Na_2_SiO_3_ 3. K_2_SiO_3_	Concentration	Activator/ Binder Ratio	Modulus	Curing Condition	Age	Shrinkage Type	Shrinkage Value (με)	Ref.
Mortar	Ambient (20 °C) and Heat 60 °C)	2	1 + 2	8% Na_2_O	0.4	1.5	Sealed Heat: 60 °C Ambient drying: 20 ± 0.5 °C, 60 ± 2% RH	14 d	Auto	20 °C-2890 60 °C-3430	[93]
Mortar	Dry heat	1 + 2 + 4	1 + 2	12 M	-	2	Sealed and oven-dried (40 °C and 80 °C)	90 d	Drying	1D 40 °C-1950 3D 40 °C-550 1D 80 °C-420 7D 80 °C-400	[94]
Concrete	Ambient, Dry heat, and Steam	1	1 + 2	8 M	-	2	Sealed 72 h-ambient 24 h-heat cured: 60 °C Dried: 23 °C, 40–60% RH	90 d	Drying	Ambien t- 1250 Heat 60 °C-100 Steam 60 °C-95	[129]
Concrete	Ambient and Dry heat	1 + 2	1 + 2	14 M	0.6	-	Sealed 24 h-ambient 24 h-heat cured: 80 °C	84 d	Drying	Ambient-729 Dry heat-538	[126]
Concrete	Dry heat	1 + 2	1 + 2	13 M	0.4	-	60 °C 80 °C 100 °C 120 °C	28 d	Drying	2453 2458 2487 2494	[124]
Concrete	Ambient and Oven	1 + 2	1 + 2	13 M	0.4 0.5 0.6 0.7	-	24 h ambient, 24 h heat, cured: 80 °C	28 d	Drying	Ambient Oven 350 420 375 460 420 500 445 540	[124]
Mortar	Water bath and Steam	1 + 2 + 5	1 + 2	16 M	0.4	-	Sealed Water bath, ambient, and 12 h-heat cured: 85 °C	180 d	Drying	Ambient-0.99% Steam-0.58%	[133]
Paste	Dry heat	1	1 + 2 + 3	8 M	0.36	-	40 °C 80 °C	120 h	Auto	40 °C-2.63% 80 °C-2.21%	[132]
Mortar	Ambient, Steam, and Carbonation	1 + 3 + 6	1 + 2	**-**	0.4	1.3	Ambient, Steam: 12 h, Carbonation (20 °C, 90% RH, 20% CO_2_ concentration)	120 d	Drying	Ambient-4460 Steam (80 °C)-4250 Steam (60 °C)-3850 Carbonation-3785.71	[95]

## Data Availability

No new data were created or analyzed in this study. Data sharing is not applicable to this article.
